# *Trueperella pyogenes* promotes the synthesis and maturation of IL-1β in murine macrophages

**DOI:** 10.3389/fimmu.2025.1614952

**Published:** 2025-06-11

**Authors:** Meimei Yang, Yunhao Hu, Junwei Wang, Wenlong Zhang, Bin Liu

**Affiliations:** ^1^ Department of Neurology, The Fourth Affiliated Hospital of Harbin Medical University, Harbin, Heilongjiang, China; ^2^ College of Veterinary Medicine, Northeast Agricultural University, Harbin, Heilongjiang, China

**Keywords:** *T. pyogenes*, PLO, NF-κB, NLRP3, caspase-1, IL-1β

## Abstract

*Trueperella pyogenes* (*T. pyogenes*) is an important opportunistic pathogen in animals and can also cause diseases in humans. Previous studies have shown that *T. pyogenes* infection can upregulate the levels of pro-inflammatory cytokines, such as interleukin-1 beta (IL-1β), in host tissues. However, the underlying mechanisms are not yet fully understood. In the current study, we found that both inactivated *T. pyogenes* cells (iTP) and pyolysin (PLO, a major virulence factor of *T. pyogenes*) can promote the transcription of the IL-1β gene both *in vivo* and *in vitro*. iTP-caused upregulation of IL-1β gene transcription is dependent on nuclear factor-kappa B (NF-κB). On the other hand, we determined that PLO, but not iTP, can promote the maturation of IL-1β by activating caspase-1-mediated processing of pro-IL-1β. Further, we confirmed that PLO can induce potassium ion (K^+^) efflux in mouse macrophages, thereby activating caspase-1 in a Nod-like receptor protein 3 (NLRP3)-dependent manner. Blocking K^+^ efflux or knocking down the expression of NLRP3 both inhibited caspase-1 activation and pro-IL-1β processing. Taken together, these findings demonstrate that *T. pyogenes* can promote IL-1β expression at both the transcriptional and post-translational levels in a murine macrophage model. These results significantly enhance our understanding of the pathogenesis of *T. pyogenes* and the interactions between *T. pyogenes* and host immune system.

## 1 Introduction

*Trueperella pyogenes* (*T. pyogenes*), formerly known as *Arcanobacterium pyogenes*, is a ubiquitous species usually found on the skin, oropharynx, and upper respiratory, urogenital, and gastrointestinal tracts of healthy animals (1, 2). When the immune system of the host animals is compromised by adverse factors, such as wound or primary infections caused by other pathogens (3, 4), this opportunistic organism can cause diseases in animals on its own or accompanied by other organisms (2, 5–7). According to a retrospective study, the most common clinical manifestations associated with *T. pyogenes* are endometritis, mastitis, abscesses, pneumonia, and lymphadenitis in domestic animals (2). Although rare, *T. pyogenes* can also infect humans and lead to diseases such as endocarditis and pharyngitis (8–10). Therefore, the diseases caused by *T. pyogenes* not only pose a threat to the domestic industry but also affect public health. Investigating the pathogenesis mechanisms of this organism may promote the development of prevention and control strategies and methods against these diseases.

The expression of various virulence factors contributes to the colonization and invasion abilities of *T. pyogenes* in various hosts (11) and the causation of various types of diseases. Studies on this organism have shown that pyolysin (PLO), collagen-binding protein A (CbpA), fimbriae (Fim), neuraminidases (NanH and NanP), and biofilm play essential roles in the pathogenesis of the bacterium (12). PLO can bind to and form pores in cholesterol-containing membranes, which grants this toxin the ability to kill host cells at high concentrations (13). CbpA and Fim serve in the adhesion and colonization of the bacterium to host tissues. CbpA can bind collagen, while the ligand for Fim has not been identified. The Nans of the organism can promote tissue colonization by reducing mucus viscosity through cleaving terminal sialic acid residues from complex glycoproteins, glycolipids, and carbohydrates on host cell receptors. The biofilm of the organism promotes resistance to adverse factors, such as antimicrobials, and attacks from the host’s immune system. Other predicted virulence factors of *T. pyogenes*, although rarely investigated, include various exoenzymes, such as serine proteases or DNAses (12). To date, our understanding of the virulence factors of *T. pyogenes* is limited, and their precise roles in pathogenesis need further study.

*T. pyogenes* is potent in eliciting inflammatory diseases. Our previous study showed that *T. pyogenes* induced the expression of interleukin-1β (IL-1β) in lung of mice and precision-cut lung slices of pig (14). *T. pyogenes* also induced the expression of IL-1β in mouse macrophage cell line (15). Since IL-1β has been confirmed to contribute to the severity of inflammatory diseases (16), the overproduction of this cytokine may be a reason for the inflammatory tissue damage of host caused by *T. pyogenes* infection.

Limited research showed that PLO, the pore-forming toxin (PFT) secreted by *T. pyogenes* (1), could enhance the production of IL-1β in mice and cultured macrophages (17) through promoting the release of IL-1β via inducing gasdermin D (GSDMD)-mediated pyroptosis (18). However, the comprehensive impact of this organism on IL-1β production is not clear yet.

The current study intends to investigate how *T. pyogenes* cells and PLO affects the production of IL-1β. Our results showed that *T. pyogenes* cells were capable of inducing the synthesis of pro-IL-1β in a NF-κb-dependent manner and PLO could promote the maturation of IL-1β by activating potassium ions (K^+^)/NLRP3 inflammasome/caspase-1 pathway in PLO-treated mouse macrophages. Our data is valuable for our understanding of the pathogenesis of *T. pyogenes*.

## 2 Materials and methods

### 2.1 Ethics statement

The experimental protocol involving animals was approved by the Ethics Committee on the Use and Care of Animals at Northeast Agricultural University (Approval No. NEAUEC20240383), in accordance with the Guide for the Care and Use of Laboratory Animals (Institute of Laboratory Animal Resources, Commission on Life Sciences, National Research Council, 2000). All experiments were conducted in compliance with the relevant guidelines and regulations.

### 2.2 Preparation of inactivated *T. pyogenes*

*T. pyogenes* (strain 0912) was isolated and stored in our laboratory and was cultured in Martin broth medium with 10% fetal bovine serum (FBS) under aerobic conditions. The culture was harvested during the late-log phase. The density of *T. pyogenes* cells was determined by colony counting method. *T. pyogenes* was inactivated by adding formaldehyde into the culture to a final concentration of 0.5% and incubating at 37°C for 48 h. The inactivation of the *T. pyogenes* cells was determined by bacterial culture in liquid cultural medium. Then, the inactivated *T. pyogenes* cells (iTP) were washed twice with ice-cold sterile phosphate buffer saline (PBS) and were finally suspended in sterile PBS. The *T. pyogenes* suspension was adjusted to a concentration of 4 × 10^10^ colony-forming units per milliliter (CFU/mL) and stored at -80°C until use.

### 2.3 Preparation and characterization of recombinant PLO and mutants

Recombinant plasmid pET-30a (+)-plo, which encodes recombinant mature PLO (rPLO) with an N-terminal extension of a 52-aa fusion peptide, was laboratory constructed (19). Recombinant plasmid pET-30a (+)-plo W497F was constructed according to another previous study (20). pET-30a (+)-plo W497F was constructed using a PCR-mediated DNA mutation system fast mutagenesis system (Transgen Biotech, Beijing). The recombinant plasmid pET-30a-plo was used as a template, and the sequences of the primers are 5′-GCAACTGGCCTAGCGTTCGATCCGTGGTGG-3′ and 3′-TGACCGGATCGCAAGCTAGGCACCACCTGG-5′. The resulting pET-30a (+)-plo W497F encodes rPLO W497F with the tryptophan (W) at the position of 497 of rPLO, which was replaced with a phenylalanine (F). The cell membrane binding capacity of rPLO W497F [named His-PLO. F_497_ in Billington, et al. (20)] was reduced compared with that of rPLO.

The recombinant plasmids were transformed into *Escherichia coli* (*E. coli*) Rosetta (DE3)™ competent cells. rPLO and rPLO W497F were expressed by inducing with isopropyl-β-D-thiogalactoside (IPTG) and then purified with nickel-charged resin. The purified proteins were dialyzed against PBS with 5% glycerol at 4°C for 48 h. The proteins were quantified by Bradford method and stored at -80°C until use.

The assays for detecting the membrane binding ability of rPLO and rPLO W497F were performed as previously described (21). Briefly, sheep red blood cell membranes (RBCM) were prepared. rPLO and rPLO W497F, or PBS (as a negative control), were incubated with the RBCM suspension for 30 minutes at 37°C and then washed twice with PBS. The proteins in the samples were separated by sodium dodecyl sulfate polyacrylamide gel electrophoresis (SDS-PAGE) and transferred onto nitrocellulose (NC) membranes. The binding of rPLO and rPLO W497F to RBCM was determined by Western blot analysis using an anti-His tag monoclonal antibody as the primary antibody.

For the hemolysis assay, the concentrations of rPLO and rPLO W497F were first adjusted to 200 μg/mL. The proteins were then subjected to two-fold serial dilutions with PBS in V-bottomed 96-well microtiter plates. An equal volume of a 2% suspension of sheep red blood cells (sRBCs) was added to each well. The mixtures were incubated at 37°C for 30 minutes. Complete hemolysis was defined as the absence of visible sRBC precipitate at the bottom of the well.

To observe the pores formed by the recombinant proteins, the concentrations of rPLO and rPLO W497F were adjusted to 400 μg/mL. Then, 100 μL of each protein (rPLO and rPLO W497F) was mixed separately with 900 μL of a 2% sRBC suspension. The mixtures were incubated at 37°C for 30 minutes, then centrifuged at 5000 r/min for 7 minutes at 4°C. The supernatant was discarded, and the precipitates were resuspended in 200 μL of fresh PBS and negatively stained. The specimens were observed under a Hitachi H-7650 electron microscope at an acceleration voltage of 100 kV.

### 2.4 Preparation of bone marrow-derived macrophages

Bone marrow-derived macrophages (BMDMs) were obtained from 3- to 6-week-old C57BL/6 mice, with slight modifications to a previously described method. After sacrificing the mice, the bone marrow was flushed from the femurs. The cells were then washed and resuspended in Roswell Park Memorial Institute-1640 (RPMI-1640) medium, supplemented with 10% FBS and 15% (v/v) L929 cell-conditioned medium, which served as a biological source of macrophage colony stimulating factor (M-CSF). The medium was replaced on days 3 and 5, and non-adherent cells were removed. The cells were used for experiments starting on day 7.

### 2.5 Determination of the sublytic concentration of rPLO against BMDMs

The sublytic concentration of rPLO against BMDMs was determined using a lactate dehydrogenase (LDH) release assay. Briefly, BMDMs were seeded into a 48-well cell plate at a density of 1×10^5^ cells per well. The cells were then incubated with various concentrations of rPLO (8, 4, 2, 1, 0.5, 0.25, and 0.125 μg/mL) in RPMI-1640 medium without FBS for 30 minutes at 37°C. For the control group, cells were treated with RPMI-1640 medium supplemented with PBS. The cells were observed and photographed using an inverted microscope. The amount of released LDH in the medium was measured using an LDH cytotoxicity assay kit (C0016, Beyotime Biotechnology) according to the manufacturer’s instructions. The sublytic concentration of rPLO was defined as the lowest concentration that did not induce significant morphological changes or cell detachment but resulted in a significant increase in LDH release compared to the control group. LDH release was also measured in cells treated with various concentrations of rPLO W497F.

### 2.6 Cytokines induction

For the *in vivo* experiment, nine 6-week-old female BALB/c mice were randomly divided into three groups. On days 0, 7, and 14, mice in the two experimental groups received subcutaneous injections of rPLO (25 μg in a final volume of 300 μL) and iTP (1 × 10^10^ CFU in a final volume of 300 μL), respectively. Mice in the control group received subcutaneous injections of PBS containing 5% glycerol (300 μL). On day 21, the mice were humanely sacrificed, and tissues near the injection sites were collected. Part of the tissues was weighed and homogenized in 5 mL sterile PBS on ice. The homogenates were centrifuged at 12,000 r/min at 4°C for 5 min, and the supernatants were harvested. The expression levels of cytokines in the supernatants were detected by enzyme-linked immunosorbent assay (ELISA). The amount of cytokines in the homogenate supernatants was converted to the amount per gram of muscle tissue for further statistical analysis. The remaining tissues were frozen in liquid nitrogen and ground into powder. Total RNA was extracted using TRIzol™ reagent (Invitrogen, USA) to measure cytokine expression at the mRNA level by quantitative real-time PCR (qRT-PCR) assays.

In the *in vivo* IL-1β blocking assay, each mouse received a mixture containing 30 μg of IL-1β-specific polyclonal antibody (Proteintech, China) and 50 μg of rPLO subcutaneously. Control mice received 50 μg of rPLO only. Two days later, the mRNA level of interleukin-6 (IL-6) in the tissues surrounding the injection sites was determined by qRT-PCR.

For the *in vitro* experiment, BMDMs were seeded in a 6-well cell plate at a density of 1×10^6^ cells per well. The cells were incubated with rPLO (0.03 μg/mL) or iTP (4 × 10^9^ CFU) in RPMI-1640 medium supplemented with 10% FBS for 16 h at 37°C. Supernatants were harvested after the incubation and stored at -80°C. The amount of cytokines in the supernatants was determined by ELISA. Total RNA was extracted from the cells using TRIzol™ reagent, and cytokine expression at the mRNA level was determined by qRT-PCR assays.

### 2.7 Electrophysiology

For patch-clamp experiments, RAW 264.7 cells were plated overnight in Dulbecco’s Modified Eagle Medium (DMEM) supplemented with 10% FBS on sterile glass coverslips in six-well plates at a density of 1×10^4^ cells per well. On the day of the experiment, the cells were incubated with or without rPLO at the sublytic concentration for 30 minutes. Whole-cell recordings of potassium ion (K^+^) currents were performed using standard patch-clamp techniques as previously described with slight modifications (22). The cells were superfused at room temperature with an extracellular solution containing (in mM): 150 NaCl, 6 KCl, 10 HEPES, 1.5 CaCl_2_, 1 MgCl_2_, and 1 ethylene glycol tetraacetic acid (EGTA) (pH 7.3). The pipette solution contained (in mM): 145 KCl, 10 HEPES, 1 MgCl_2_, and 1 EGTA (pH 7.3). Voltage steps ranging from -40 mV to +120 mV were applied from a holding potential of -60 mV to determine the current-voltage (I-V) relationship. Each pulse lasted for 500 ms with an interval of 5 s. All recordings were performed at least 5 minutes after establishing the whole-cell configuration. Currents were recorded using an Axon 700B Amplifier (Axon Instruments, Foster City, CA). Data were filtered at 2 kHz and digitized at 10 kHz. A CED Power 1401 Analogue/Digital Interface (Cambridge Electronic Design Limited, Cambridge, United Kingdom), controlled by Signal 3.06 software (Cambridge Electronic Design Limited), was used for generating voltage commands, recording the current, and collecting data. Data were analyzed offline using Origin v8.0 (Northampton, MA). Currents were normalized to cell capacitance (pA/pF). Five to six cells were recorded for each experimental condition. All recordings were performed within a 1-hour window after exposing the cells to the recombinant proteins.

### 2.8 Determination of intracellular potassium (K^+^) concentrations

BMDMs were seeded in a six-well cell culture plate at a density of 1×10^6^ cells per well and were subsequently treated with rPLO at the sublytic concentration, rPLO W497F (at a concentration equal to that of rPLO), nigericin (5 mM), and rPLO plus 120 mM KCl at 37°C for 30 minutes. The intracellular K^+^ concentrations were then determined using a cellular potassium concentration quantitative test kit (chemical colorimetric) (HL70032.2, Shanghai Haling Biological Technology Co., Ltd.) according to the manufacturer’s instructions.

### 2.9 Small interfering RNA silencing of NOD-like receptor protein 3 gene in J774A.1 cells

J774A.1 cells were transduced with pooled Nlrp3 siRNA/shRNA/RNAi lentivirus (mouse) (iV043412, Abm Inc.) or scrambled siRNA GFP lentivirus (LVP015-G, Abm Inc.). The cells were transduced with Nlrp3 siRNA or control siRNA and were selected using puromycin. The efficiency of NLRP3 silencing was confirmed by Western blot analysis.

### 2.10 Cell treatment and western blot assay

Cells were treated with iTP or lipopolysaccharides (LPS) (1 μg/mL) for various durations to examine the expression of pro-IL-1β. In some experiments, cells were co-treated with iTP plus TPCA-1 (a NF-κB pathway inhibitor, 1 μM) or BAY11-7082 (a NF-κB pathway inhibitor, 10 μM).

The culture medium was discarded, and a fresh medium without FBS but containing rPLO, rPLO W497F, or nigericin was added. Cells were then incubated at 37°C for 30 min. In some experiments, cells treated with rPLO were also exposed to KCl (5, 10, 25, 50, 100, or 120 mM), NaCl (125 mM), Z-VAD-FMK (a pan-caspase inhibitor, 20 μM), Z-YVAD-FMK (a caspase-1 inhibitor, 20 μM), or Z-DEVD-FMK (a caspase-3 inhibitor, 20 μM).

After treatment, cells were lysed with a cell lysis buffer for Western blotting and IP (Beyotime, China) supplemented with PMSF. The culture supernatant components were precipitated by adding trichloroacetic acid. The precipitates were then re-dissolved in NaOH. The samples were denatured in 5× reducing sample buffer for SDS-PAGE and boiled for 10 min. Proteins separated by SDS-PAGE were transferred to NC or polyvinylidene difluoride membranes and immunoblotted with primary antibodies against caspase-1 p20 (B200721, Biolegend), NLRP3 (AG-20B-0014, Adipogen), IL-1β (12507S, Cell Signaling Technology), and GAPDH (TA-08, ZSGB-BIO). The membranes were then treated with HRP-linked secondary antibodies: anti-rabbit (ZDR-5306, ZSGB-BIO), anti-rat (ZB-2307, ZSGB-BIO), or anti-mouse (ZDR-5307, ZSGB-BIO).

### 2.11 Caspases enzymatic activity measurement

BMDMs were seeded in 24-well cell cultural plate at a density of 2 × 10^5^ cells per well. BMDMs was primed with or without LPS and then treated with rPLO. Active caspase-1, caspase-3, and caspase-8 was quantified by using caspase activity assay Kit (caspase-1 C1102; caspase-3 C1116; caspase-8 C1152, Beyotime Biotechnology) according to the manufacturer’s instructions.

### 2.12 qRT-PCR

Total cellular RNA was reverse-transcribed into cDNA using a reverse transcriptase kit. qRT-PCR was performed using the SYBR Green method. The specific primer sequences for mouse IL-1β, interleukin-6 (IL-6), interleukin-10 (IL-10), tumor necrosis-α factor (TNF-α), and β-actin are listed in **Table 1**. Expression levels were normalized to that of β-actin. All measurements were performed in triplicate, and expression changes were normalized to untreated controls.

**Table 1:** The primers used to perform qRT-PCR.

Target	Forward Primer	Reverse Primer
β-actin	GGAGGGGGTTGAGGTGTT	GTGTGCACTTTTATTGGTCTCAA
IL-1β	GGGCCTCAAAGGAAAGAATC	TCTTCTTTGGGTATTGCTTGG
TNF-α	ATAGCTCCCAGAAAAGCAAGC	CACCCCGAAGTTCAGTAGACA
IL-6	CTGCAAGAGACTTCCATCCAG	AGTGGTATAGACAGGTCTGTTGG
IL-10	CTCCCCTGTGAAAATAAGAGC	GCCTTGTAGACACCTTGGTC

### 2.13 ELISA

The amount of TNF-α, IL-1β, IL-6 and IL-10 in samples were detected by commercial ELISA kits from Uscn Life Science Inc., China (TNF-α, SEA133Mu; IL-1β, SEA563Mu; IL-6, SEA079Mu; IL-10, SEA056Mu) according to the manufacturers’ instructions.

### 2.14 Statistical analysis

All statistical analyses were performed based on data acquired from three or five biological replications of the experiment. One-way or two-way AVONA test was used to analyze the data using Graphpad Prism 10.4.0 software (GraphPad Software), the normality of the data distribution was determined by Shapiro-Wilk test. The results are presented as mean ± SD. A *p* value < 0.05 was considered significant (* *p* < 0.05, ** *p* < 0.01, *** *p* < 0.001, **** *p* < 0.0001).

## 3 Results

### 3.1 Characterization of rPLO and rPLO W497F

**Figure 1:**
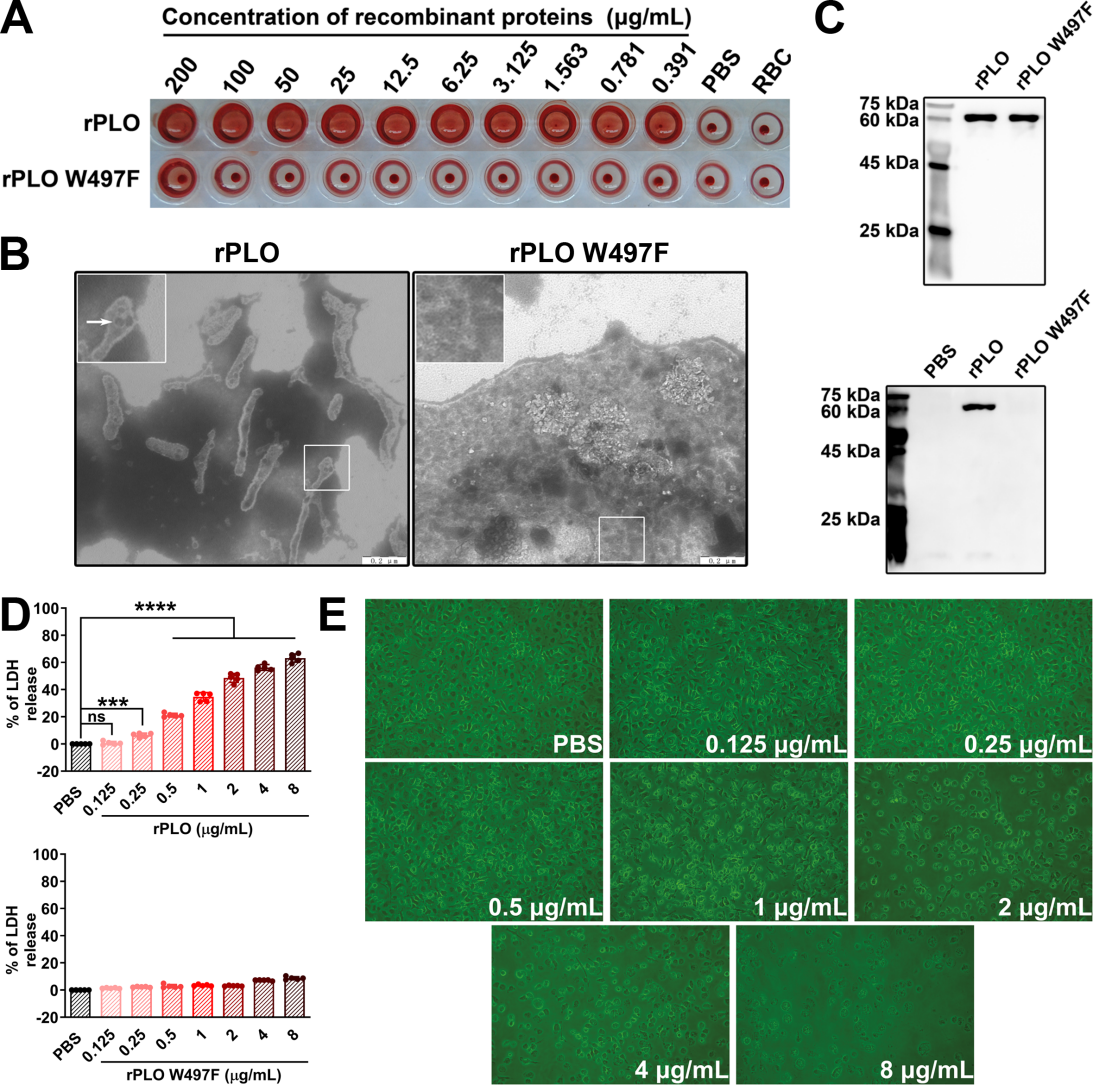
Characterization of rPLO and rPLO W497F **(A)** The lowest concentration of rPLO to cause complete hemolysis of sRBCs were 3.125 μg/mL, while rPLO W497F could not cause complete hemolysis even at the highest concentration (200 μg/mL). **(B)** Electron microscope observation showed that rPLO formed pores in cell membrane of sRBCs, while rPLO W497F did not. (scale bar=0.2 μm) **(C)** The upper panel showed the purified rPLO and rPLO W497F. The lower panel showed that rPLO, but not rPLO W497F, could bind RBCM. **(D)** LDH assays showed that rPLO at concentrations higher than 0.125 μg/mL could elicit significant LDH release from BMDMs (upper panel), and rPLO W497F did not cause significant LDH release in BMDMs even at the concentration as high as 8 μg/mL (lower panel). (The data was obtained from five biological replicates. One-way ANOVA test was used for analysis. *** indicates p<0.001, **** indicates p<0.0001, ns indicates no significant difference) **(E)** rPLO at concentrations lower than 1 μg/mL did not cause significant morphological changes of BMDMs. (200×).

The purified rPLO and rPLO W497F were serially diluted and then incubated with RBCs to determine the differences in their hemolytic activities. The results showed that a concentration of 3.125 µg/mL of rPLO was sufficient to cause complete hemolysis, whereas rPLO W497F only caused partial hemolysis even at a concentration as high as 200 µg/mL (**Figure 1A**). These results suggested that rPLO W497F had almost completely lost hemolytic activity. TEM observations revealed that rPLO could form pores in the membranes of RBCs, whereas rPLO W497F could not (**Figure 1B**). RBCM binding assays further showed that rPLO could bind to RBCM, while rPLO W497F could not (**Figure 1C**).

When incubated with BMDMs, rPLO at concentrations higher than 0.25 µg/mL caused significant LDH release (p < 0.01) from BMDMs within a 30-min incubation period (**Figure 1D**, upper panel). In contrast, rPLO W497F did not cause significant LDH release even at a concentration as high as 8 µg/mL (**Figure 1D**, lower panel). Microscopic observations indicated that rPLO at concentrations up to 0.5 µg/mL did not induce obvious morphological changes or exfoliation in BMDMs (**Figure 1E**). These results suggested that 0.5 µg/mL of rPLO could disrupt the cell membrane integrity of BMDMs without causing cell death within 30 min. Therefore, 0.5 µg/mL was considered a sub-cytotoxic concentration of rPLO for BMDMs and was used in subsequent experiments.

### 3.2 iTP and PLO induce the expression proinflammatory cytokines *in vivo* and *in vitro*

**Figure 2:**
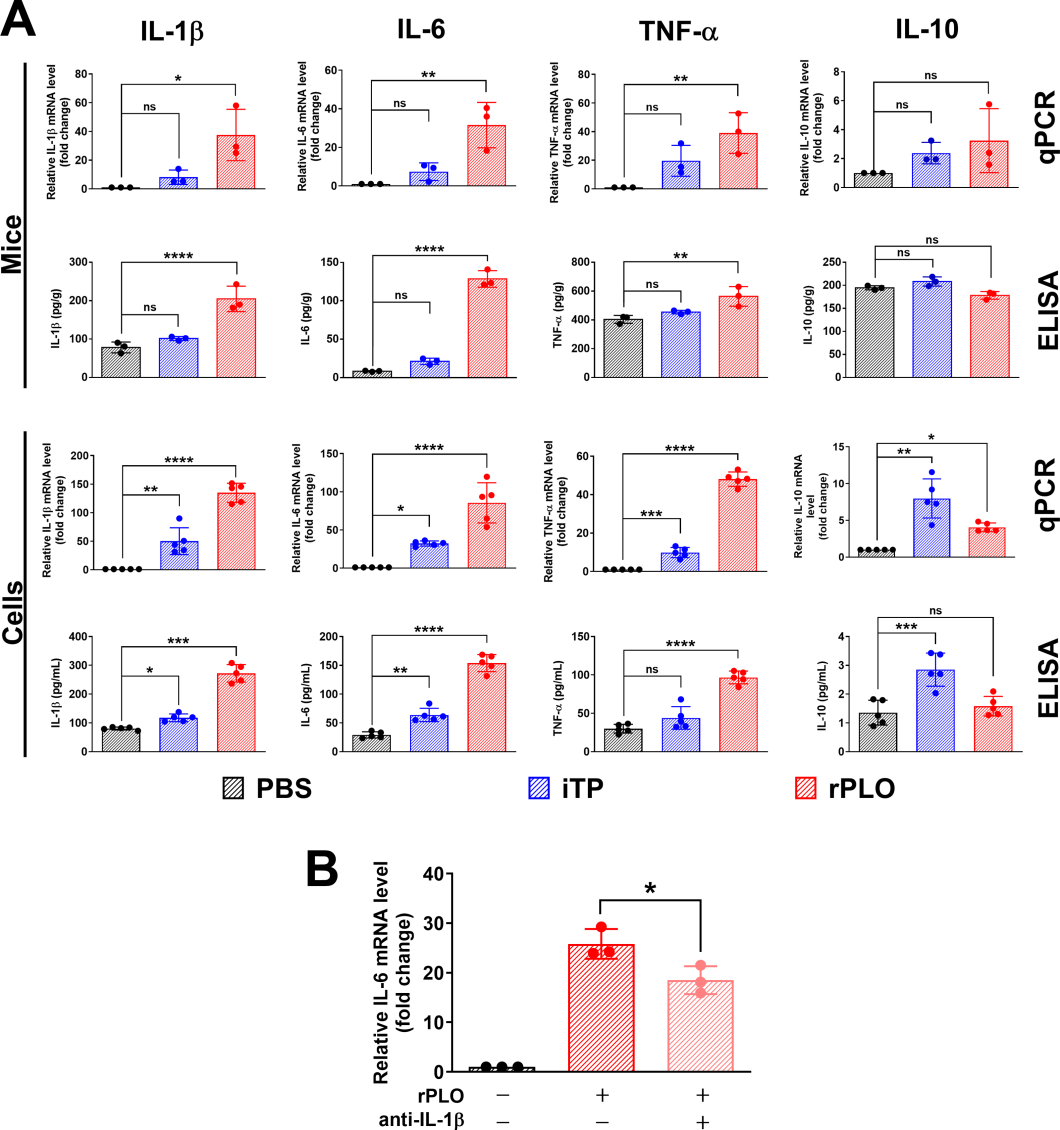
iTP and rPLO affects the production of inflammation-associated cytokines in mice and BMDMs at mRNA and protein levels. **(A)** rPLO treatment significantly upregulated the expression of IL-1β, IL-6 and TNF-α at mRNA and protein levels but did not affect the expression of IL-10. iTP slightly affected the expression of IL-1β, IL-6 and TNF-α but significantly upregulated the expression of IL-10 (The data was obtained from three biological replicates of *in vivo* experiment and from five biological replicates of *in vitro* experiment. One-way ANOVA test was used for analysis. * indicates p < 0.05, ** indicates p<0.01, *** indicates p<0.001, **** indicates p<0.0001, ns indicates no significant difference) **(B)** IL-1β-specific antibody co-treatment significantly inhibited the synthesis of IL-1β mRNA in rPLO-challenged mice. (The data was obtained from three biological replicates. One-way ANOVA test was used for analysis. * indicates p < 0.05).

iTP and purified rPLO were used to treat mice and BMDMs, respectively. qPCR and ELISA results showed that rPLO treatment significantly upregulated the levels of IL-1β, TNF-α, and IL-6 at both the mRNA and protein levels in the muscles of mice and in BMDMs. While not as potent as rPLO treatment, iTP treatment also increased the expression levels of IL-1β, TNF-α, IL-6, and IL-10 in both mice and cells (**Figure 2A**). Injection of anti-IL-1β-specific antibody with rPLO significantly inhibited the synthesis of IL-6 mRNA in the tissues of rPLO-challenged mice (**Figure 2B**). Since IL-1β can induce the expression of IL-6, this result indicates that IL-1β plays an important role in the occurrence of PLO-induced inflammation.

### 3.3 iTP induces the synthesis of pro-IL-1β in a NF-κB–dependent manner

**Figure 3:**
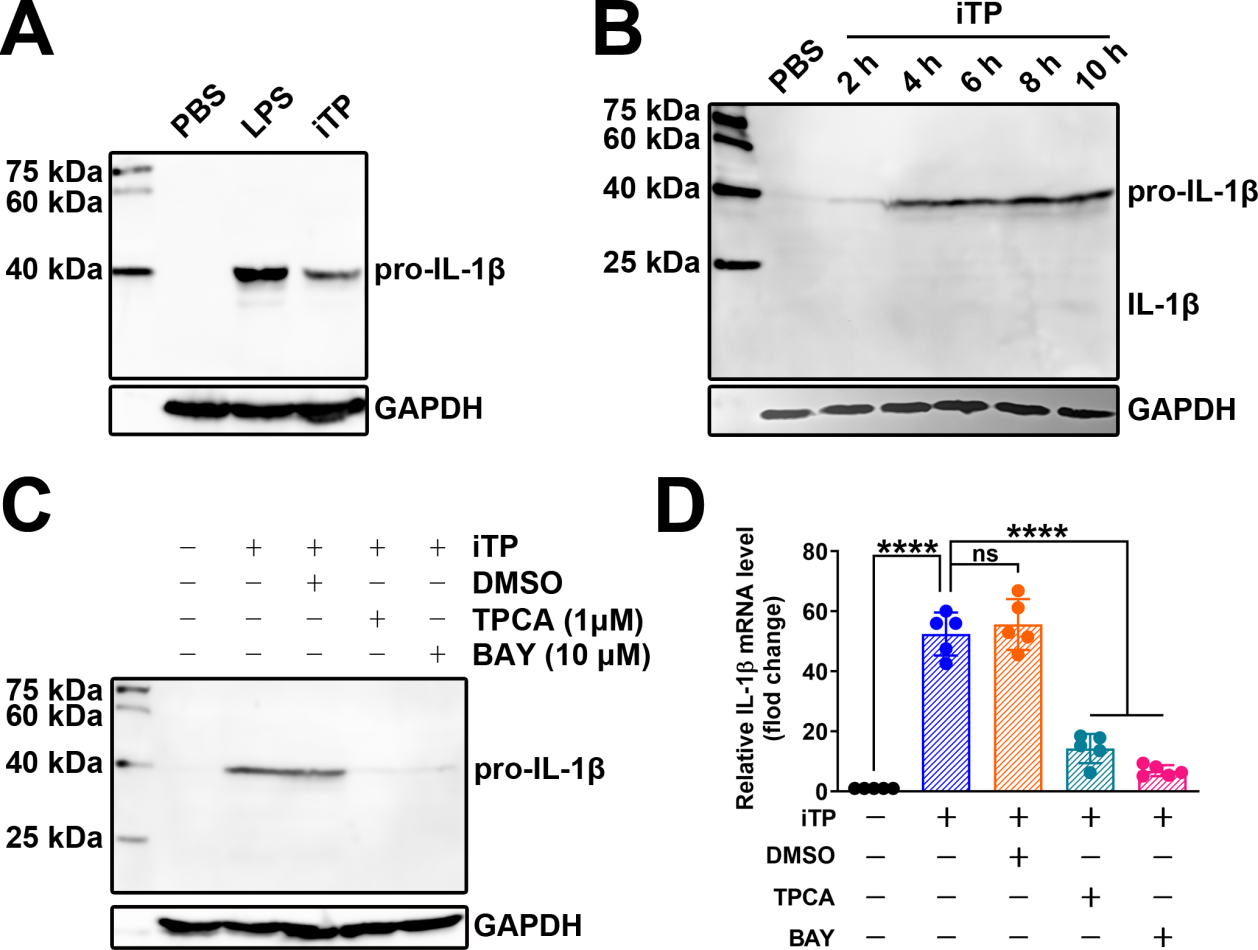
iTP induces the synthesis of pro-IL-1β in BMDMs in a NF-κB-dependent manner **(A)** Both LPS and iTP could induce the synthesis of pro-IL-1β in BMDMs. **(B)** 2-h incubation with iTP was enough for inducing the synthesis of pro-IL-1β in BMDMs. **(C)** TPCA-1 (TPCA) (1 μM) and BAY11-7082 (BAY) (10 μM) significantly inhibited the synthesis of pro-IL-1β caused by iTP as determined by western blot assay. **(D)** TPCA and BAY also most completely abolished the transcription of IL-1β mRNA caused by iTP as determined by qRT-PCR. (The data was obtained from five biological replicates. One-way ANOVA test was used for analysis. **** indicates p<0.0001, ns indicates no significant difference).

Western blot assays showed that iTP could induce the synthesis of pro-IL-1β in BMDMs (**Figure 3A**). LPS, a classical TLR4 agonist, was used as a positive control (**Figure 3A**). A 2-h treatment of BMDMs with iTP was sufficient to induce pro-IL-1β synthesis (**Figure 3B**). However, iTP was not efficient in promoting the maturation of IL-1β, as the mature IL-1β (the 17 kDa band) could only be detected in BMDMs incubated with iTP for 10 h, and the 17 kDa band on the NC membrane was very weak (**Figure 3B**). TPCA-1 and BAY11-7082, two inhibitors of the NF-κB pathway, significantly inhibited the iTP-induced synthesis of pro-IL-1β at both the protein (**Figure 3C**) and mRNA levels (**Figure 3D**). These results indicate that iTP induces the synthesis of pro-IL-1β in a NF-κB–dependent manner.

### 3.4 PLO promotes the rapid maturation of IL-1β in BMDMs

**Figure 4:**
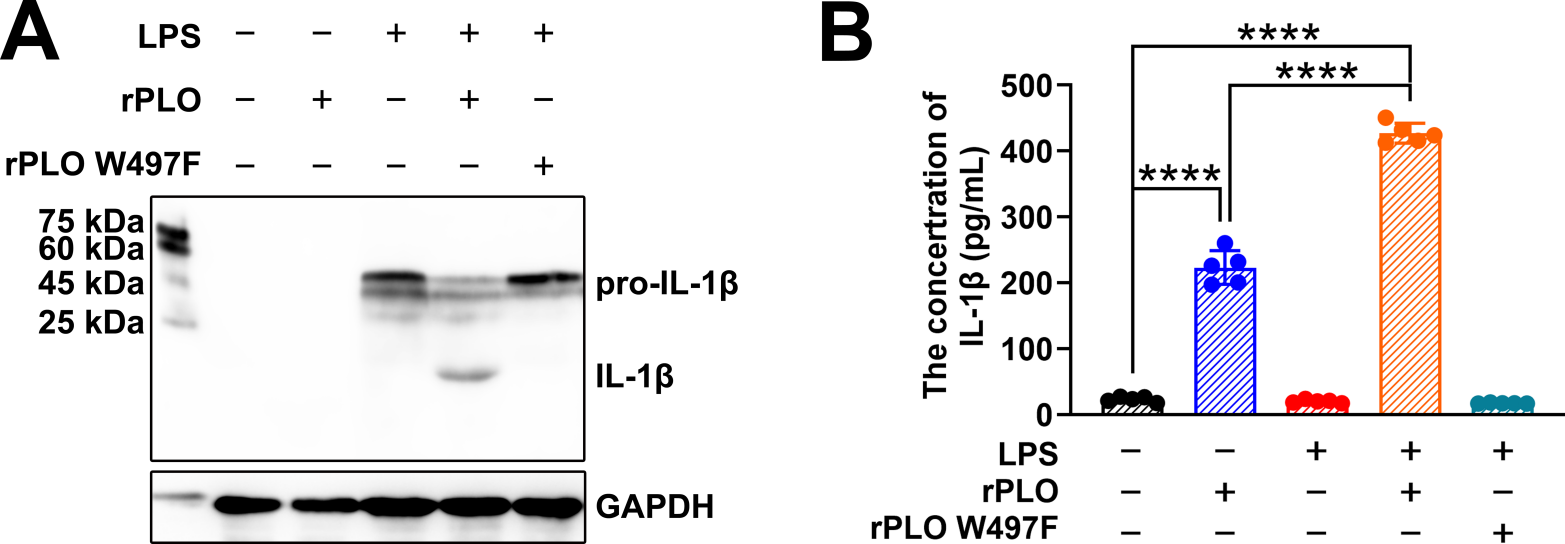
rPLO induces pro-IL-1β processing in BMDMs. **(A)** Western blot assay showed that rPLO caused pro-IL-1β processing in LPS-primed BMDMs after incubating for 30 min, while rPLO W497F treatment did not lead to pro-IL-1β processing in LPS-primed BMDMs. **(B)** The concentration of IL-1β in cultural supernatant of BMDMs after the treatment as determined by ELISA. The results showed that rPLO could promote the release of IL-1β in both LPS-primed and -unprimed BMDMs. However, priming with LPS significantly increased the quantity of IL-1β in cultural supernatant after rPLO treatment compared to treating cell with rPLO only. (The data was obtained from five biological replicates. One-way ANOVA test was used for analysis. **** indicates p<0.0001).

BMDMs were primed with LPS for 4 h and then incubated with rPLO or rPLO W497F. Western blot assays showed that pretreatment with LPS promoted the synthesis of pro-IL-1β but did not induce the maturation of IL-1β in BMDMs (**Figure 4A**). Subsequent incubation with rPLO led to the production of mature IL-1β (as shown by the 17 kDa band on the NC membrane) within 30 min (**Figure 4A**). In contrast, rPLO W497F failed to elicit the maturation of IL-1β in LPS-primed BMDMs (**Figure 4A**). ELISA results revealed that the amount of IL-1β in the culture supernatants of cells treated with rPLO was significantly higher (p < 0.01) than that in the untreated controls, regardless of whether the cells were primed with LPS (**Figure 4B**). The IL-1β production in LPS-primed cells was significantly higher than that in unprimed cells after treatment with rPLO (p < 0.01) (**Figure 4B**). By contrast, the IL-1β production in cells treated with LPS + rPLO W497F did not significantly differ from that in untreated cells (**Figure 4B**). These findings suggested that a 30-min incubation with rPLO could induce rapid maturation of IL-1β and the cell membrane binding and pore-forming activities of rPLO are essential for promoting pro-IL-1β maturation in BMDMs.

### 3.5 PLO-induced IL-1β maturation requires caspase-1

**Figure 5:**
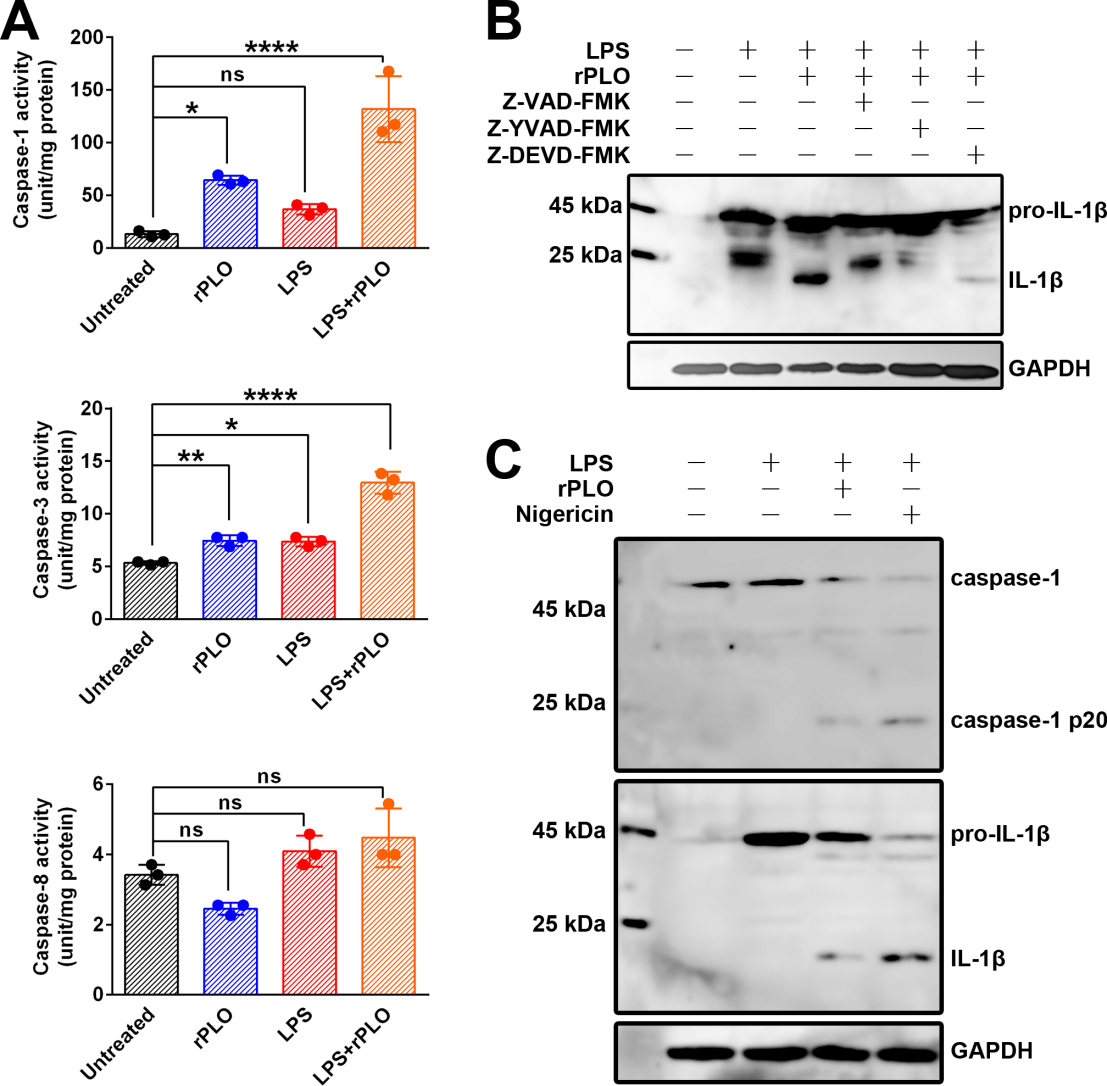
rPLO induces pro-IL-1β processing by activating caspase-1 in BMDMs **(A)** Measurement of the enzymatic activity of caspase-1, caspase-3, and caspase-8 in treated cells. The results showed that rPLO itself could significantly activate caspase-1 and caspase-3 but not caspase-8. LPS treatment caused slightly increase in caspase-1 and caspase-3 enzymatic activity. Priming with LPS could further enhance the enzymatic activity of caspase-1 and caspase-3 in rPLO-treated cells. (The data was obtained from three biological replicates. One-way ANOVA test was used for analysis. * indicates p < 0.05, ** indicates p < 0.01, **** indicates p< 0.0001, ns indicates no significant difference) **(B)** Z-VAD-FMK (pan-caspase inhibitor) and Z-YVAD-FMK (caspase-1 inhibitor) completely abolished the pro-IL-1β processing in LPS+rPLO-treated cells. In contrast, Z-DEVD-FMK (caspase-3 inhibitor) did not completely inhibit LPS+rPLO-caused pro-IL-1β processing. **(C)** rPLO and nigricin caused caspase-1 activation and pro-IL-1β processing in LPS-primed cells.

Enzymatic activity measurements showed that rPLO treatment activated caspase-1 and caspase-3, but not caspase-8, within 30 min (**Figure 5A**). To determine which caspase is responsible for the maturation of IL-1β, cells were treated with rPLO in the presence of caspase inhibitors. The results showed that Z-VAD-FMK (a pan-caspase inhibitor) and Z-YVAD-FMK (a caspase-1 inhibitor) completely abolished the maturation of IL-1β, whereas Z-DEVD-FMK (a caspase-3 inhibitor) did not (**Figure 5B**). To confirm that caspase-1 was activated in rPLO-treated cells, LPS-primed BMDMs were treated with rPLO or nigericin. The results showed that rPLO treatment indeed activated caspase-1 within 30 min (**Figure 5C**). Therefore, caspase-1 is responsible for PLO-induced IL-1β maturation.

### 3.6 PLO-induced K+ efflux is responsible for the activation of caspase-1

**Figure 6:**
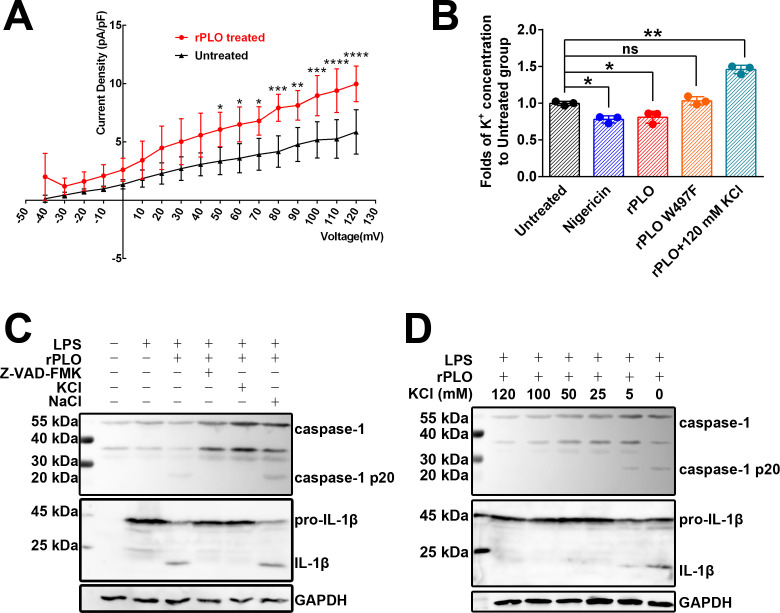
K^+^ efflux plays essential role in rPLO-induced caspase-1 activation and pro-IL-1β processing in BMDMs. **(A)** rPLO treatment caused increase in outward K^+^ current in Raw 264.7 cells. The experiment has been repeated for 5 times. (The data was obtained from five biological replicates. Two-way ANOVA test was used for analysis. * indicates p < 0.05, ** indicates p < 0.01, *** indicates p< 0.001, **** indicates p< 0.0001) **(B)** Change of intracellular K^+^ concentration in BMDMs subjecting different treatments. The intracellular K^+^ concentration of untreated BMDMs was set as a standard. The results were showed as the fold of intracellular K^+^ concentrations of the BMDMs receiving different treatments to intracellular K^+^ concentration of the untreated BMDMs. (The data was obtained from three biological replicates. Two-way ANOVA test was used for analysis. One-way ANOVA test was used for analysis. * indicates p < 0.05, ** indicates p < 0.01, ns indicates no significant difference) **(C)** Extracellular KCl, but not NaCl, inhibited the activation of caspase-1 and pro-IL-1β processing induced by rPLO in LPS-primed BMDMs. Z-VAD-FMK served as a positive control. The result indicated that extracellular K+, but not extracellular Na+, exhibited the inhibitory effect to the activation of caspase-1 and pro-IL-1β processing in rPLO+LPS-treated BMDMs. **(D)** The inhibition effect of caspase-1 activation and pro-IL-1β processing by KCl at various concentrations in rPLO+LPS-treated BMDMs. The results showed that supplement with 5 mM KCl in the cultural medium caused partial inhibition of caspase-1 activation and pro-IL-1β processing. KCl at concentrations higher than 5 mM completely abolished the rPLO+LPS-caused caspase-1 activation and pro-IL-1β processing.

Since K^+^ plays an essential role in caspase-1 activation (23), we first evaluated the K^+^ flux in rPLO-treated macrophages using electrophysiological techniques. As shown in **Figure 6A**, exposure of RAW 264.7 cells to rPLO significantly increased the whole-cell outward K^+^ current density at depolarizing potentials. Next, we assessed the intracellular K^+^ concentrations in BMDMs treated with recombinant proteins. The results showed that BMDMs treated with rPLO (p < 0.05) or nigericin (p < 0.01), a well-characterized K^+^ ionophore, exhibited significant reductions in intracellular K^+^ concentration compared to untreated cells, whereas BMDMs treated with rPLO W497F had intracellular K^+^ concentrations similar to those of untreated BMDMs (**Figure 6B**). BMDMs treated with rPLO plus 120 mM KCl showed increased intracellular K^+^ concentrations compared to untreated cells (**Figure 6B**), indicating a reversal of intracellular K^+^ depletion in rPLO-treated cells in the presence of high extracellular K^+^ concentrations.

Western blot assays revealed that 120 mM KCl, but not 120 mM NaCl, inhibited the activation of caspase-1 and the processing of pro-IL-1β induced by rPLO in LPS-primed BMDMs (**Figure 6C**). Even at a low extracellular KCl concentration of 5 mM, the activation of caspase-1 and processing of pro-IL-1β induced by rPLO in LPS-primed BMDMs were partially inhibited (**Figure 6D**).

The above results indicate that PLO can efficiently induce K^+^ efflux in macrophages, leading to caspase-1 activation and pro-IL-1β processing.

### 3.7 PLO-induced caspase-1 activation and pro-IL-1β processing in macrophages are NLRP3-dependent

**Figure 7:**
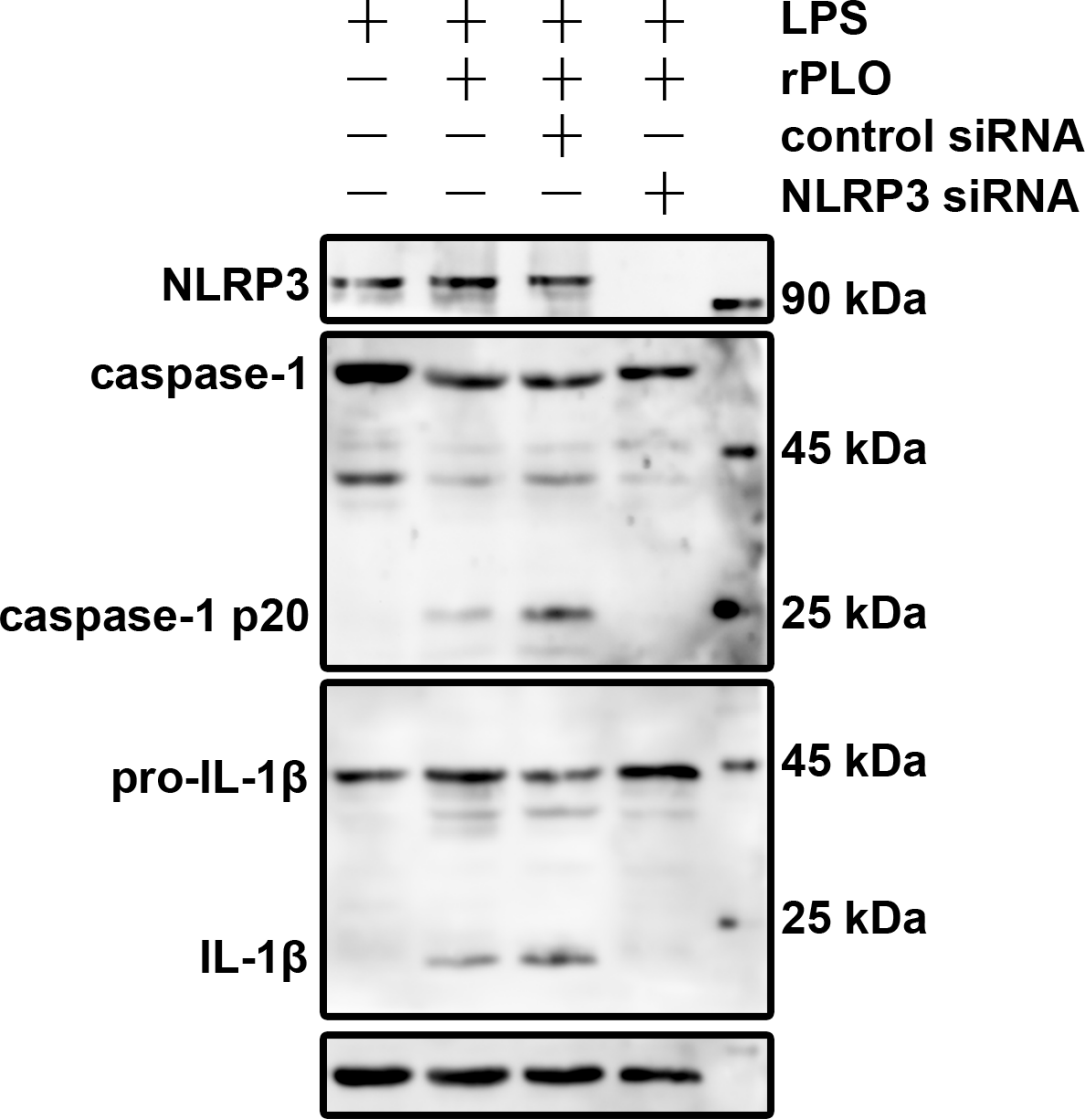
NLRP3 plays essential role in rPLO+LPS-induced caspase-1 activation and pro-IL-1β processing in J774A.1 cells Knockdown of NLRP3 in J774A.1 cells using specific siRNA was verified by Western blot analysis. The cells were then treated accordingly. The results showed that rPLO+LPS-induced caspase-1 activation and pro-IL-1β processing in J774A.1 cells were completely abolished in the absence of NLRP3.

We first constructed J774A.1 cell lines expressing either nlrp3 siRNA or control siRNA via lentivirus-mediated transduction. The cells were then treated as described. **Figure 7** shows that rPLO induced caspase-1 activation and pro-IL-1β processing in J774A.1 cells expressing control siRNA, but not in those expressing nlrp3 siRNA. These results indicate that rPLO-induced caspase-1 activation and pro-IL-1β processing are dependent on the NLRP3 inflammasome.

## 4 Discussion

*T. pyogenes* infection causes the overexpression of various cytokines, among which IL-1β is one of the most potent in promoting inflammation. Inflammation is a double-edged sword for hosts. On the positive side, it strengthens the immune response against pathogens. On the negative side, excessive inflammation often induces host tissue injury and complicates diseases. Some mechanisms used by pathogens for survival or immune escape can affect the inflammatory process in hosts, thereby worsening the disease (24). A recent study showed that *Salmonella* exploits IL-1β signaling to outcompete commensal microbes and establish gut colonization in mice (25). This study also showed that the absence of IL-1β attenuated *Salmonella*-caused tissue damage by impairing neutrophil recruitment to the gut (25). The upregulation of IL-1β expression by *T. pyogenes* may similarly facilitate the establishment of infection, as a study showed that intrauterine infusion of *T. pyogenes* resulted in a significant increase in the percentage of polymorphonuclear neutrophils in the uterus samples of challenged cows (26). Therefore, uncovering the underlying mechanisms of *T. pyogenes*-induced IL-1β expression is essential for understanding the pathogenesis of this organism.

Mammals have developed a two-step system for IL-1β expression. First, pro-IL-1β, the cytosolic precursor to IL-1β, is produced. Second, pro-IL-1β is proteolytically cleaved (maturation of IL-1β) and released from the cells (27). These two steps are tightly controlled under physiological conditions. Disturbing these steps can lead to uncontrolled IL-1β expression and excessive inflammation. The current study confirmed that *T. pyogenes* affects IL-1β expression at both steps. Based on our observations, *T. pyogenes* cells were efficient in inducing the synthesis of IL-1β mRNA and pro-IL-1β protein (**Figures 3A, B**). However, *T. pyogenes* cells were not as potent in inducing the maturation of IL-1β. Unlike *T. pyogenes* cells, PLO, the PFT secreted by *T. pyogenes*, was capable of promoting IL-1β maturation. A brief exposure (30 min) to a sublytic concentration (0.5 μg/mL) of rPLO was sufficient to activate the proteolysis of pro-IL-1β in BMDMs (**Figure 4A**). The cooperation between bacterial cell components and the secreted PFT efficiently accelerates the production of mature IL-1β.

The synthesis of pro-IL-1β has been shown to be driven by transcription factors such as NF-κB, AP-1, and HIF-1 (16). The current study demonstrated that the synthesis of pro-IL-1β in macrophages stimulated with iTP could be significantly inhibited by NF-κB pathway inhibitors (**Figure 3C**). This finding suggests that NF-κB plays an essential role in *T. pyogenes*-induced IL-1β production. Therefore, drugs targeting the NF-κB pathway could be valuable for alleviating the severity of diseases caused by *T. pyogenes*. Further study of us will focus on the receptor/s that can recognize cellular components of *T. pyogenes* and also the cellular components in *T. pyogenes* cells that stimulate the synthesis of pro-IL-1β in macrophages.

PLO can form pores in cell membranes (**Figure 1B**), which disrupts the barrier between intracellular and extracellular compartments and facilitates ion movement across the cell membrane. The concentration of intracellular K^+^ is significantly higher than that of extracellular K^+^ (28). The PLO pores in the cell membrane may provide conduits for outward K^+^ movement, following the principle of free diffusion. The current study confirmed the efflux of K^+^ in rPLO-treated macrophages using the patch-clamp technique and colorimetric detection, which showed an increased outward K^+^ current (**Figure 6A**) and a decreased intracellular K^+^ concentration (**Figure 6B**), respectively. The depletion of intracellular K^+^ can activate the NLRP3 inflammasome pathway, thereby activating caspase-1 (23). Therefore, we further investigated the impact of rPLO-induced K^+^ loss on the activation of caspase-1. The results confirmed that supplementing the culture medium with KCl effectively inhibited the activation of caspase-1 and the maturation of IL-1β (**Figures 6C, D**).

Furthermore, we investigated the role of the NLRP3 inflammasome in rPLO-induced caspase-1 activation and IL-1β maturation by knocking down the expression of NLRP3 in macrophages. This is because the NLRP3 inflammasome plays an important role in pathogen sensing and controls the post-translational processing of pro-inflammatory cytokines, such as IL-1β (29). Upon activation, NLRP3 oligomerizes with the adaptor protein apoptosis-associated speck-like protein containing a CARD (ASC) and pro-caspase-1, leading to the autocatalytic activation of caspase-1, which in turn processes pro-IL-1β to produce active cytokines (30). Our results showed that the absence of NLRP3 protein completely abolished the activation of caspase-1 and the maturation of IL-1β in rPLO-treated macrophages (**Figure 7**). These results suggest that the K^+^/NLRP3/caspase-1 pathway plays an essential role in the maturation of IL-1β.

IL-1β possesses multiple and diverse properties in response to infection, injury, and immune challenges (31). However, pro-IL-1β does not exhibit activity (16). In addition, it has been reported that mature IL-1β molecules are preferentially released through GSDMD pores (32). *T. pyogenes* manipulates the maturation step of IL-1β by secreting PLO, which may facilitate the functional activation and release of the cytokine. This property of *T. pyogenes* may cause a rapid accumulation of IL-1β in host’s tissues, thereby worsening the infection by inducing excessive inflammation. Although PFTs can cause tissue damage by killing cells directly at high concentrations, *in vivo* host cells are probably exposed to sublytic concentrations of these toxins (33). Therefore, the ability of PLO to induce IL-1β maturation and release may explain its role *in vivo*.

Macrophages contribute to the early stages of innate defense against infections (34, 35). Meanwhile, pathogens can infect or manipulate macrophages to facilitate their escape from the immune attack of the host’s immune system or the establishment of infection (36, 37). The relationship between pathogens and macrophages is still a fascinating area for investigation. Macrophages can secrete various cytokines upon stimulation. Previous studies have shown that M1 macrophages mainly secrete pro-inflammatory cytokines such as IL-1β, IL-6, and TNF-α (38, 39). These properties make macrophages an ideal model for investigating the mechanisms of pro-inflammatory cytokine production in response to *T. pyogenes* challenge. Therefore, the current study employed primary bone marrow-derived macrophages (BMDMs) and macrophage cell lines (RAW264.7 and J774A.1 cells) as models to investigate the impact of *T. pyogenes* on the expression of IL-1β. BMDMs were used in most of the experiments in the current study because they are more similar to wild-type macrophages than RAW264.7 and J774A.1 macrophage cell lines. RAW264.7 cells were used to measure the rPLO-induced K^+^ current. Our previous studies showed that PLO treatment elicits GSDMD-mediated pyroptosis, which may compromise the accuracy of K^+^ current measurements by forming GSDMD pores in the cell membrane. A previous study showed that RAW264.7 cells lack ASC molecules, which are essential for the activation of caspase-1 and subsequent GSDMD-mediated pyroptosis (40). Therefore, measuring the K^+^ current in the rPLO-treated RAW264.7 model may more accurately reflect the K^+^ current changes induced by rPLO. J774A.1 cells were used to generate an NLRP3 knockdown cell line because J774A.1 cells can proliferate *in vitro*, which facilitates the subsequent screening for obtaining recombinant cells. According to the study, J774A.1 cells express both NLRP3 and ASC, the two molecules essential for the activation of caspase-1 (40). This makes J774A.1 cells a good model for investigating NLRP3 inflammasome-mediated caspase-1 activation and IL-1β maturation.

**Figure 8:**
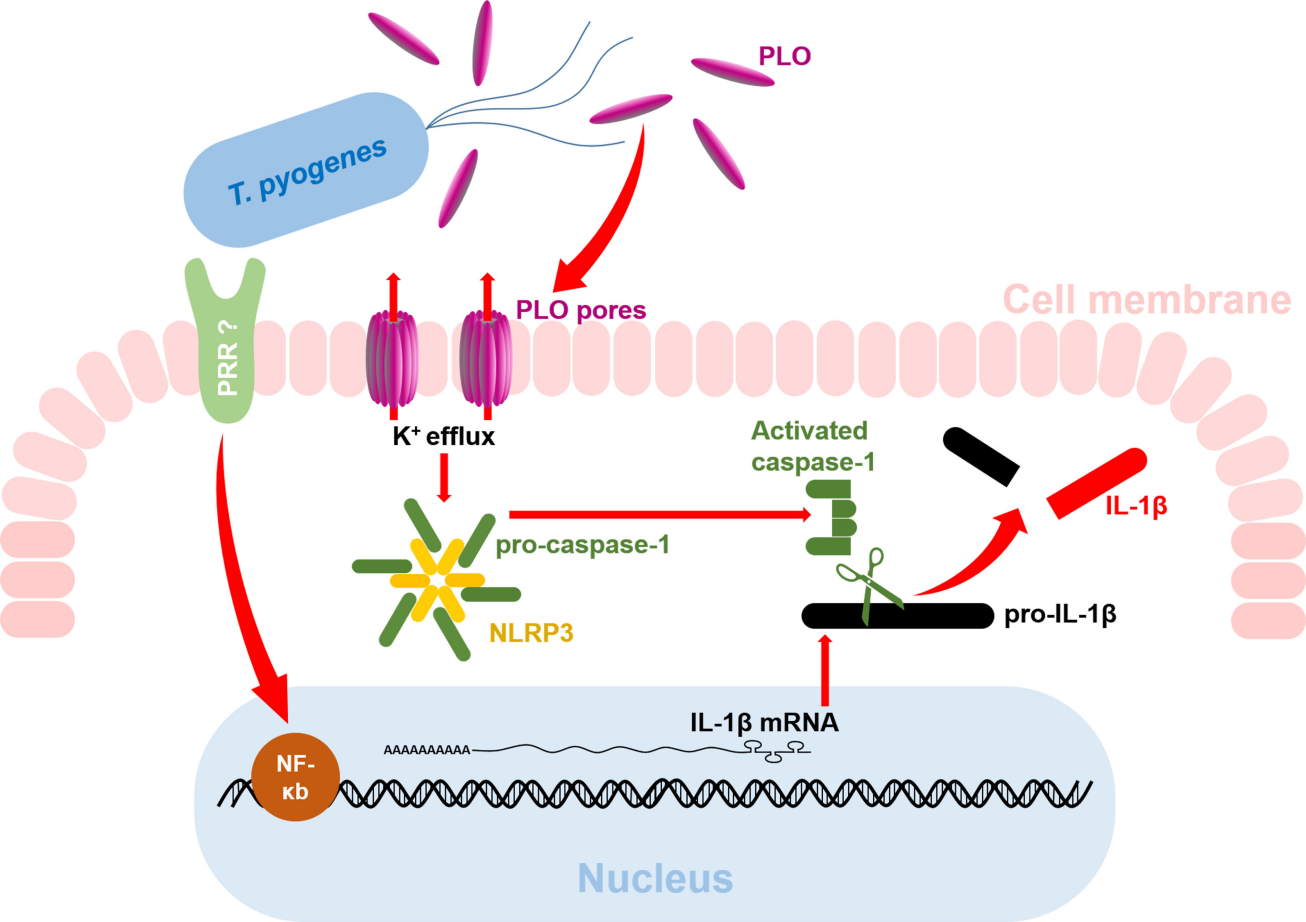
Diagram of the mechanism of *T. pyogenes*-induced IL-1β production in macrophages. The red arrows indicate the findings from the current study. The question marks indicate areas that remain unknown and require further investigation.

In conclusion, our results indicate that *T. pyogenes* can boost the production of mature IL-1β at both the transcriptional and post-translational levels (**Figure 8**). Mechanistically, cellular components of *T. pyogenes* can promote the synthesis of IL-1β mRNA by activating the NF-κB pathway, thereby causing the accumulation of pro-IL-1β in macrophages. Subsequently, PLO can efficiently activate caspase-1 via the K^+^/NLRP3 pathway, thereby promoting the maturation of IL-1β. Our findings shed light on the pathogenesis of *T. pyogenes*.

## Data Availability

The original contributions presented in the study are included in the article/supplementary material. Further inquiries can be directed to the corresponding authors.

## References

[1] Jost BH Billington SJ. Arcanobacterium pyogenes: molecular pathogenesis of an animal opportunist. Antonie Van Leeuwenhoek. (2005) 88:87–102. doi: 10.1007/s10482-005-2316-5 16096685

[2] Ribeiro MG Risseti RM Bolanos CA Caffaro KA de Morais AC Lara GH. Trueperella pyogenes multispecies infections in domestic animals: a retrospective study of 144 cases (2002 to 2012). Vet Q. (2015) 35:82–7. doi: 10.1080/01652176.2015.1022667 25793626

[3] Panciera RJ Confer AW. Pathogenesis and pathology of bovine pneumonia. Vet Clin North Am Food Anim Pract. (2010) 26:191–214. doi: 10.1016/j.cvfa.2010.04.001 20619179 PMC7185769

[4] Confer AW. Update on bacterial pathogenesis in BRD. Anim Health Res Rev. (2009) 10:145–8. doi: 10.1017/S1466252309990193 20003651

[5] Belser EH Cohen BS Keeler SP Killmaster CH Bowers JW Miller KV. Epethelial presence of Trueperella pyogenes predicts site-level presence of cranial abscess disease in white-tailed deer (Odocoileus virginianus). PloS One. (2015) 10:e0120028. doi: 10.1371/journal.pone.0120028 25803047 PMC4372218

[6] Eisenberg T Nagib S Hijazin M Alber J Lammler C Hassan AA. Trueperella pyogenes as cause of a facial abscess in a grey slender loris (Loris lydekkerianus nordicus)–a case report. Berl Munch Tierarztl Wochenschr. (2012) 125:407–10. 23045803

[7] Ishiyama D Mizomoto T Ueda C Takagi N Shimizu N Matsuura Y. Factors affecting the incidence and outcome of Trueperella pyogenes mastitis in cows. J Vet Med Sci. (2017) 79:626–31. doi: 10.1292/jvms.16-0401 PMC5383188 28163273

[8] Marchionatti E Kittl S Sendi P Perreten V. Whole genome-based antimicrobial resistance, virulence, and phylogenetic characteristics of Trueperella pyogenes clinical isolates from humans and animals. Vet Microbiol. (2024) 294:110102. doi: 10.1016/j.vetmic.2024.110102 38749210

[9] Stuby J Lardelli P Thurnheer CM Blum MR Frei AN. Trueperella pyogenes endocarditis in a Swiss farmer: a case report and review of the literature. BMC Infect Dis. (2023) 23:821. doi: 10.1186/s12879-023-08810-y 37996784 PMC10668470

[10] Meili Z. Trueperella pyogenes pharyngitis in an immunocompetent 40-year-old man. BMJ Case Rep. (2020) 13:e236129. doi: 10.1136/bcr-2020-236129 PMC7684657 33229475

[11] Wen X Cheng J Liu M. Virulence factors and therapeutic methods of Trueperella pyogenes: A review. Virulence. (2025) 16:2467161. doi: 10.1080/21505594.2025.2467161 39983010 PMC11849936

[12] Rzewuska M Kwiecien E Chrobak-Chmiel D Kizerwetter-Swida M Stefanska I Gierynska M. Pathogenicity and virulence of trueperella pyogenes: A review. Int J Mol Sci. (2019) 20:2737. doi: 10.3390/ijms20112737 31167367 PMC6600626

[13] Yang L Liang H Wang B Ma B Wang J Zhang W. Evaluation of the potency of two pyolysin-derived recombinant proteins as vaccine candidates of trueperella pyogenes in a mouse model: pyolysin oligomerization and structural change affect the efficacy of pyolysin-based vaccines. Vaccines (Basel). (2020) 8:79. doi: 10.3390/vaccines8010079 32050696 PMC7157609

[14] Qin L Meng F He H Li S Zhang H Sun Y. Inflammation plays a critical role in damage to the bronchiolar epithelium induced by Trueperella pyogenes *in vitro* and in *vivo* . Infect Immun. (2023) 91:e0027323. doi: 10.1128/iai.00273-23 37929972 PMC10714949

[15] Huang T Cui K Song X Jing J Lin J Wang X. MTOR involved in bacterial elimination against Trueperella pyogenes infection based on mice model by transcriptome and biochemical analysis. Vet Microbiol. (2019) 235:199–208. doi: 10.1016/j.vetmic.2019.06.021 31383303

[16] Bent R Moll L Grabbe S Bros M. Interleukin-1 beta-A friend or foe in Malignancies? Int J Mol Sci. (2018) 19:2155. doi: 10.3390/ijms19082155 30042333 PMC6121377

[17] Zhang W Wang H Wang B Zhang Y Hu Y Ma B. Replacing the 238th aspartic acid with an arginine impaired the oligomerization activity and inflammation-inducing property of pyolysin. Virulence. (2018) 9:1112–25. doi: 10.1080/21505594.2018.1491256 PMC6086297 30067143

[18] Liang H Wang B Wang J Ma B Zhang W. Pyolysin of trueperella pyogenes induces pyroptosis and IL-1beta release in murine macrophages through potassium/NLRP3/caspase-1/gasdermin D pathway. Front Immunol. (2022) 13:832458. doi: 10.3389/fimmu.2022.832458 35371034 PMC8965163

[19] Hu Y Zhang W Bao J Wu Y Yan M Xiao Y. A chimeric protein composed of the binding domains of Clostridium perfringens phospholipase C and Trueperella pyogenes pyolysin induces partial immunoprotection in a mouse model. Res Vet Sci. (2016) 107:106–15. doi: 10.1016/j.rvsc.2016.04.011 27473983

[20] Billington SJ Songer JG Jost BH. The variant undecapeptide sequence of the Arcanobacterium pyogenes haemolysin, pyolysin, is required for full cytolytic activity. Microbiology. (2002) 148:3947–54. doi: 10.1099/00221287-148-12-3947 12480898

[21] Yan M Hu Y Bao J Xiao Y Zhang Y Yang L. Isoleucine 61 is important for the hemolytic activity of pyolysin of Trueperella pyogenes. Vet Microbiol. (2016) 182:196–201. doi: 10.1016/j.vetmic.2015.11.031 26711048

[22] Thomas J Epshtein Y Chopra A Ordog B Ghassemi M Christman JW. Anthrax lethal factor activates K(+) channels to induce IL-1beta secretion in macrophages. J Immunol. (2011) 186:5236–43. doi: 10.4049/jimmunol.1001078 PMC3583536 21421849

[23] Munoz-Planillo R Kuffa P Martinez-Colon G Smith BL Rajendiran TM Nunez G. K(+) efflux is the common trigger of NLRP3 inflammasome activation by bacterial toxins and particulate matter. Immunity. (2013) 38:1142–53. doi: 10.1016/j.immuni.2013.05.016 PMC3730833 23809161

[24] Dewamitta SR Nomura T Kawamura I Hara H Tsuchiya K Kurenuma T. Listeriolysin O-dependent bacterial entry into the cytoplasm is required for calpain activation and interleukin-1 alpha secretion in macrophages infected with Listeria monocytogenes. Infect Immun. (2010) 78:1884–94. doi: 10.1128/IAI.01143-09 PMC2863535 20194588

[25] Zigdon M Sawaed J Zelik L Binyamin D Ben-Simon S Asulin N. Salmonella manipulates the host to drive pathogenicity via induction of interleukin 1beta. PloS Biol. (2024) 22:e3002486. doi: 10.1371/journal.pbio.3002486 38236896 PMC10826948

[26] McDougall S Graham EM Aberdein D Reed CB Burke CR. Development of an intrauterine infection model in the postpartum dairy cow. N Z Vet J. (2022) 70:22–31. doi: 10.1080/00480169.2021.1950069 34185614

[27] Barker BR Taxman DJ Ting JP. Cross-regulation between the IL-1beta/IL-18 processing inflammasome and other inflammatory cytokines. Curr Opin Immunol. (2011) 23:591–7. doi: 10.1016/j.coi.2011.07.005 PMC3380339 21839623

[28] Kettritz R Loffing J. Potassium homeostasis - Physiology and pharmacology in a clinical context. Pharmacol Ther. (2023) 249:108489. doi: 10.1016/j.pharmthera.2023.108489 37454737

[29] Karmakar M Katsnelson M Malak HA Greene NG Howell SJ Hise AG. Neutrophil IL-1beta processing induced by pneumolysin is mediated by the NLRP3/ASC inflammasome and caspase-1 activation and is dependent on K+ efflux. J Immunol. (2015) 194:1763–75. doi: 10.4049/jimmunol.1401624 PMC4369676 25609842

[30] Subramanian N Natarajan K Clatworthy MR Wang Z Germain RN. The adaptor MAVS promotes NLRP3 mitochondrial localization and inflammasome activation. Cell. (2013) 153:348–61. doi: 10.1016/j.cell.2013.02.054 PMC3632354 23582325

[31] Piccioli P Rubartelli A. The secretion of IL-1beta and options for release. Semin Immunol. (2013) 25:425–9. doi: 10.1016/j.smim.2013.10.007 24201029

[32] Xia S Zhang Z Magupalli VG Pablo JL Dong Y Vora SM. Gasdermin D pore structure reveals preferential release of mature interleukin-1. Nature. (2021) 593:607–11. doi: 10.1038/s41586-021-03478-3 PMC8588876 33883744

[33] Aroian R van der Goot FG. Pore-forming toxins and cellular non-immune defenses (CNIDs). Curr Opin Microbiol. (2007) 10:57–61. doi: 10.1016/j.mib.2006.12.008 17234446

[34] Jonsson S Musher DM Chapman A Goree A Lawrence EC. Phagocytosis and killing of common bacterial pathogens of the lung by human alveolar macrophages. J Infect Dis. (1985) 152:4–13. doi: 10.1093/infdis/152.1.4 3874252

[35] Franke-Ullmann G Pfortner C Walter P Steinmuller C Lohmann-Matthes ML Kobzik L. Characterization of murine lung interstitial macrophages in comparison with alveolar macrophages in *vitro* . J Immunol. (1996) 157:3097–104. doi: 10.4049/jimmunol.157.7.3097 8816420

[36] Anand N Sehgal R Kanwar RK Dubey ML Vasishta RK Kanwar JR. Oral administration of encapsulated bovine lactoferrin protein nanocapsules against intracellular parasite Toxoplasma gondii. Int J Nanomedicine. (2015) 10:6355–69. doi: 10.2147/IJN.S85286 PMC4605239 26504384

[37] Anand N Kanwar RK Dubey ML Vahishta RK Sehgal R Verma AK. Effect of lactoferrin protein on red blood cells and macrophages: mechanism of parasite-host interaction. Drug Des Devel Ther. (2015) 9:3821–35. doi: 10.2147/DDDT.S77860 PMC4524381 26251568

[38] Anand N Peh KH Kolesar JM. Macrophage repolarization as a therapeutic strategy for osteosarcoma. Int J Mol Sci. (2023) 24:2858. doi: 10.3390/ijms24032858 36769180 PMC9917837

[39] Schweer D Anand N Anderson A McCorkle JR Neupane K Nail AN. Human macrophage-engineered vesicles for utilization in ovarian cancer treatment. Front Oncol. (2022) 12:1042730. doi: 10.3389/fonc.2022.1042730 36713536 PMC9875020

[40] Hirano S Zhou Q Furuyama A Kanno S. Differential regulation of IL-1beta and IL-6 release in murine macrophages. Inflammation. (2017) 40:1933–43. doi: 10.1007/s10753-017-0634-1 28766178

